# FK506-binding protein 10 (FKBP10) regulates lung fibroblast migration via collagen VI synthesis

**DOI:** 10.1186/s12931-018-0768-1

**Published:** 2018-04-19

**Authors:** Larissa Knüppel, Katharina Heinzelmann, Michael Lindner, Rudolf Hatz, Jürgen Behr, Oliver Eickelberg, Claudia A. Staab-Weijnitz

**Affiliations:** 1 Comprehensive Pneumology Center, Ludwig-Maximilians-Universität and Helmholtz Zentrum Munich, Max-Lebsche-Platz 31, 81377 Munich, Germany; 2 Member of the German Center of Lung Research (DZL), Munich, Germany; 3 Asklepios Fachkliniken Munich-Gauting, Munich, Germany; 4 Thoraxchirurgisches Zentrum, Klinik für Allgemeine-, Viszeral-, Transplantations-, Gefäß- und Thoraxchirurgie, Klinikum Großhadern, Ludwig-Maximilians-Universität, Munich, Germany; 5 Medizinische Klinik und Poliklinik V, Klinikum der Ludwig-Maximilians-Universität, Munich, Germany; 6 Colorado Anschutz Medical Campus, Pulmonary and Critical Care Medicine University, Denver, Colorado USA

**Keywords:** FKBP10, FKBP65, migration, focal adhesion, collagen VI, lung fibrosis, fibroblast, fibulin

## Abstract

**Background:**

In idiopathic pulmonary fibrosis (IPF), fibroblasts gain a more migratory phenotype and excessively secrete extracellular matrix (ECM), ultimately leading to alveolar scarring and progressive dyspnea. Here, we analyzed the effects of deficiency of FK506-binding protein 10 (FKBP10), a potential IPF drug target, on primary human lung fibroblast (phLF) adhesion and migration.

**Methods:**

Using siRNA, FKBP10 expression was inhibited in phLF in absence or presence of 2ng/ml transforming growth factor-β1 (TGF-β1) and 0.1mM 2-phosphoascorbate. Effects on cell adhesion and migration were monitored by an immunofluorescence (IF)-based attachment assay, a conventional scratch assay, and single cell tracking by time-lapse microscopy. Effects on expression of key players in adhesion dynamics and migration were analyzed by qPCR and Western Blot. Colocalization was evaluated by IF microscopy and by proximity ligation assays.

**Results:**

FKBP10 knockdown significantly attenuated adhesion and migration of phLF. Expression of collagen VI was decreased, while expression of key components of the focal adhesion complex was mostly upregulated. The effects on migration were 2-phosphoascorbate-dependent, suggesting collagen synthesis as the underlying mechanism. FKBP10 colocalized with collagen VI and coating culture dishes with collagen VI, and to a lesser extent with collagen I, abolished the effect of FKBP10 deficiency on migration.

**Conclusions:**

These findings show, to our knowledge for the first time, that FKBP10 interacts with collagen VI and that deficiency of FKBP10 reduces phLF migration mainly by downregulation of collagen VI synthesis. The results strengthen FKBP10 as an important intracellular regulator of ECM remodeling and support the concept of FKBP10 as drug target in IPF.

## Background

Patients suffering from idiopathic pulmonary fibrosis (IPF), a highly progressive interstitial lung disease, have a median survival prognosis of 2-5 years after diagnosis [1]. The pathogenic processes are not completely understood. It is currently believed that the fibrotic response is caused by repeated micro-injuries of the respiratory epithelium [2] which leads to the release of profibrotic mediators like transforming growth factor β1 (TGF-β1), followed by myofibroblast differentiation, increased fibroblast migration, and, ultimately, excessive deposition of extracellular matrix (ECM) in the alveolar region [3–6]. More recent evidence suggests that the composition of the ECM strongly affects fibroblast phenotypes and therefore plays a crucial role in disease progression [4, 7, 8].

Aberrant fibroblast adhesion and migration are common features of fibrosis [9–11] and targeting fibroblast migration, *e.g.* by inhibition of focal adhesion kinase (FAK) or of integrins, has been proposed as a treatment strategy [12, 13]. For instance, myofibroblasts possess an increased ability to adhere to the ECM, which is mediated by focal adhesions (FA) attaching the actin cytoskeleton to the matrix [14]. Cell attachment to the ECM via clustering of integrins leads to the recruitment of numerous FA proteins with adapter, structural, and enzymatic functions [15, 16]. For instance, structural proteins like talin, vinculin and α-actinin facilitate the connection between integrins and actin fibers and provide the basis for the transmission of mechanical forces between cell and ECM [17]. To enable cell migration, turnover of FA is necessary. Several factors are involved in FA disassembly, like actin dynamics, FAK and Src phosphorylation, and ERK/MAP kinase-mediated activation of calpain-2, a calcium-dependent protease [15].

Finally, migration is strongly influenced by topology and composition of the ECM including integrin ligands like collagen, fibronectin (FN), and laminin [7]. Collagen type VI appears to play a particularly important role in this context, as several studies indicate a central, albeit context-dependent and tissue-specific role of collagen VI for migration and adhesion [18–20].

FK506-binding protein 10 (FKBP10, also termed FKBP65), a member of the family of immunophilins, is an endoplasmic reticulum (ER) -resident peptidyl prolyl isomerase and a collagen I chaperone [21]. We have previously reported upregulation of FKBP10 in experimental lung fibrosis and IPF, where it is mainly expressed by (myo)fibroblasts [22]. Deficiency of FKBP10 by siRNA-mediated knockdown in primary human lung fibroblasts (phLF) reduced the expression of profibrotic markers like α-smooth muscle actin (α-SMA), FN and collagen I, and suppressed collagen secretion [22].

As properties of the ECM play an important role in adhesion dynamics and FKBP10 has been identified previously as a regulator of collagen biosynthesis in phLF, the aim of this study was to assess the effect of FKBP10 deficiency on adhesion and migration in phLF. To elucidate the underlying mechanisms, we analyzed the effect of siRNA-mediated knockdown of FKBP10 on intracellular and membrane-spanning components of the FA complex, on regulatory events of FA turnover, on proteins involved in actin dynamics, and, finally, on a selection of ECM proteins with important emerging functions in migration.

## Methods

### Material

Primers were obtained from MWG Eurofins (Ebersberg, Germany) and are shown in Table 1. Table 2 contains used primary antibodies. HRP-linked and fluorescent labeled secondary antibodies were purchased from GE Healthcare Life Sciences (Freiburg, Germany).

**Table 1:** Primer table for Real-Time Quantitative Reverse-Transcriptase PCR (qRT-PCR). Primers were synthesized by MWG Eurofins (Ebersberg, Germany).

Target	Species	Forward primer (5′-3′)	Reverse primer (5′-3′)
CAPNS1	human	GACGCTACTCAGATGAAAGT	TCTTTGTCAAGAGATTTGAAG
CAV1	human	TCACTGTGACGAAATACTG	CGTAGATGGAATAGACACG
COL6A1	human	GACGCACTCAAAAGCA	ATCAGGTACTTATTCTCCTTCA
COL6A2	human	AGAAAGGAGAGCCTGCGGAT	AGGTCTCCCTCACGTAGGTC
COL6A3	human	CTCTACCGAGCCCAGGTGTT	ATGAGGGTGCGAACGTACTG
CORO1C	human	GTTAACAAATGTGAGATTGC	TGGAAAAGGTCAGACTTC
DHX8	human	TGACCCAGAGAAGTGGGAGA	ATCTCAAGGTCCTCATCTTCTTCA
ERK1	human	TTCGAACATCAGACCTACT	AGGTCCTGCACAATGTAG
FBLN1C	human	GCCCTGAGAACTACCG	GAGAGGTGGTAGTAGGTTATTC
FKBP10	human	CGACACCAGCTACAGTAAG	TAATCTTCCTTCTCTCTCCA
ITGB1	human	TTACAAGGAGCTGAAAAACT	AAAATGACTTCTGAGGAAAG
TLN1	human	GCTCTTTCTGTCAGATGAT	CATAGTGTCCCCATTTC

**Table 2:** Primary antibodies used in Western Blot analysis, Immunofluorescence and Proximity Ligation Assays

Target	Abbreviation	Antibody	Provider	Application
β-actin	ACTB	HRP-conjugated anti-ACTB antibody	Sigma Aldrich, St. Louis, USA	WB
Calpain-4	CAPNS1	mouse monoclonal anti-Calpain-4	Abnova, Taipei City, Taiwan	WB, IF, PLA
Caveolin-1	CAV1	rabbit monoclonal anti-Caveolin-1 antibody	Cell Signaling, Boston, USA	WB
Collagen VI α1	COL6A1	mouse monoclonal anti-Collagen VI A1 antibody	Santa Cruz, Dallas, USA	WB, IF, PLA
Collagen VI α3	COL6A3	mouse monoclonal anti-Collagen VI A3 antibody	Santa Cruz, Dallas, USA	IF, PLA
Coronin 1C	CORO1C	mouse monoclonal anti-CORO1C antibody	Santa Cruz, Dallas, USA	WB, IF, PLA
Extracellular Signaling Related Kinase 1	ERK1	mouse monoclonal anti-ERK1 antibody	BD Biosciences, New Jersey, USA	WB
ER protein 57 (Protein disulfide-isomerase A3)	ERp57	mouse monoclonal anti-ERp57	Abcam, Cambridge, UK	PLA
Fibulin-1	FBLN1	mouse monoclonal anti-FBLN1 antibody	Santa Cruz, Dallas, USA	WB, IF, PLA
FK506-binding protein 10	FKBP10	rabbit polyclonal anti-FKBP10 antibody	ATLAS, Stockholm, Sweden	WB, IF, PLA
Focal Adhesion Kinase	FAK	rabbit polyclonal anti-FAK antibody	Santa Cruz, Dallas, USA	WB
Golgin97	CDF4	mouse monoclonal anti-Golgin97 antibody	Invitrogen, Carlsbad, USA	PLA
mouse IgG (neg. ctrl)	mouse IgG	mouse IgG1κ isotype control	eBioscience, San Diego, USA	PLA
Integrin-β1	ITGB1	mouse monoclonal anti-ITGB1 antibody	Abcam, Cambridge, UK	WB
Phosphorylated Extracellular Signaling Related Kinase 1/2	p-ERK1/2	rabbit monoclonal anti-pERK1/2 (Thr202/Tyr204)	Cell Signaling, Boston, USA	WB
Phosphorylated Focal Adhesion Kinase	p-FAK Y397	rabbit monoclonal anti-pFAK (Tyr397)	Cell Signaling, Boston, USA	WB
Phosphorylated Focal Adhesion Kinase	p-FAK Y566/577	rabbit monoclonal anti-pFAK (Tyr576/Tyr577)	Biomol, Hamburg, Germany	WB
Phosphorylated SRC Proto-Oncogene, Non-Receptor Tyrosine Kinase	p-Src	rabbit polyclonal anti-pSrc (Tyr416)	Cell Signaling, Boston, USA	WB
SRC Proto-Oncogene, Non-Receptor Tyrosine Kinase	Src	mouse monoclonal anti-Src	Cell Signaling, Boston, USA	WB
Talin 1	TLN1	mouse monoclonal anti-TLN1	Sigma Aldrich, Dt. Louis, USA	WB, IF, PLA

Secondary HRP-linked antibodies and secondary antibodies (Alexa Fluor 488 goat anti-mouse IgG, Alexa Fluor 568 goat anti-rabbit IgG) for IF were purchased from GE Healthcare Life Sciences (Freiburg, Germany). 4',6-Diamidino-2-phenylindole (DAPI) was used for nuclear staining (Sigma-Aldrich, St. Louis, USA)

### Statistical Analysis

Statistical analysis was performed in GraphPadPrism 5 (GraphPad Software, San Diego, CA, USA). Results are shown as mean ± SEM. Paired t-test was used for statistical analysis. Notably, analysis using a Wilcoxon signed rank test yielded very similar results except for the scratch assays shown in Fig. 5a where results just failed significance (not shown). Significance is indicated as follows: *p<0.05, **p<0.01, ***p<0.001.

### Human Lung Material, Isolation and Culture of phLF

Primary human lung fibroblasts (phLF) were isolated from human lung tissue and derived from in total eight different patients. The tissue derived from human lung explant material of IPF patients or histologically normal regions adjacent to resected lung tumors was obtained from the BioArchive CPC-M for lung diseases at the Comprehensive Pneumology Center (CPC Munich, Germany). The study was approved by the local ethics committee of the Ludwig-Maximilians University of Munich, Germany, and all participants gave written informed consent. Isolation and culture of phLF was performed as described previously [22, 23]. Notably, in previous studies, we have never seen consistent expression differences between control and IPF fibroblasts, neither in terms of basal and TGF-β1-induced gene expression of collagens and collagen biosynthetic enzymes, nor in terms of collagen secretion [22, 23].

### Transfection of phLF and TGF-β1 Treatment

Cells were seeded at a density of 20.000–25.000 cells/cm^2^. Reverse transfection was carried out with human small interfering RNA of FKBP10 (siRNA) (s34171; Life Technologies, Carlsbad, CA) or negative control siRNA. Twenty-four hours later starvation for another 24 hours in Dulbecco’s modified Eagle medium/F-12 including 0.5% fetal bovine serum and 0.1 mM 2-phospho-L-ascorbic acid was performed. Then, cells were treated with 2 ng/ml TGF-β1 (R&D Systems, Minneapolis, MN) in starvation medium for another 24 - 48 h, followed by harvesting for RNA and protein analysis. Unless stated otherwise, all data is derived from eight independent knockdown experiments in at least three and maximally in eight different human primary fibroblast lines. For fibroblast lines that had been used for more than one knockdown experiment in different passages, typically a mean was formed for these experiments prior to statistical analysis to avoid overrepresentation of one biological replicate in the data.

### RNA Isolation and Real-Time Quantitative Reverse-Transcriptase PCR (qRT-PCR) Analysis

RNA isolation and qRT-PCR analysis were performed as described previously [22, 23].

### Protein Isolation and Western Blot analysis

Protein isolation and Western Blot analysis were performed as described previously [22, 23].

### Cell Fixation and Immunofluorescent Stainings

Cells were seeded on FN-coated coverslips (6 μg/mL, Sigma-Aldrich, St. Louis, USA), followed by serum starvation and TGF-β1 treatment for 48h as described above. The fixation method was chosen accordingly to the used antibodies. For methanol fixation, cells were washed once in 1x phosphate-buffered saline (PBS), followed by fixation with 100% methanol for 2 minutes on ice and three additional washing steps with 1x PBS to remove the residual methanol.

For para-formaldehyde (PFA) fixation, cells were washed once with 1x PBS and 4% PFA was added to the cells followed by incubation at room temperature for 20 minutes. Residual PFA was removed by three washing steps with 1x PBS. Staining of the cover slips was performed as described before [22]. Immunofluorescence (IF) was examined by acquiring z-stack images with an Axio Imager M2 Microscope (Carl Zeiss, Jena, Germany) and analysed by AxioVision 4.8 software.

### Proximity Ligation Assay (PLA)

Cells were seeded, treated with TGF-β1 for 48h, and methanol-fixed as described above. The Duolink® PLA Kit (Sigma Aldrich, St. Louis, USA) was used and carried out according to the manufacturer’s protocol. Interaction of FKBP10 with target proteins was visualized using an Axio Imager M2 Microscope (Carl Zeiss, Jena, Germany).

### Cell Adhesion Assay

For analysis of cell attachment in FKBP10-deficient phLF, cells were seeded and treated as described above. After 48h of TGF-β1 treatment and 96h of siRNA knockdown, cells were trypsinized and counted. Per condition, four replicates with 100.000 cells per 48-well were seeded and incubated for 1 hour at 37°C, 5% CO_2_. Wells were carefully washed once with 1 x PBS to remove non-adherent cells and attached cells were fixed with 4% PFA as described before. Cells were stained with 4′,6-Diamidin-2-phenylindol (DAPI) and Phalloidin, labeled with Alexa Fluor 568 (Invitrogen) followed by imaging using an LSM T-PMT microscope (Carl Zeiss, Jena, Germany). Attached cells were counted using IMARIS Software 9.0. Results were normalized to non-treated control and visualized as percentage of attached cells.

### Scratch Assay

Cells were seeded at a density of 35.000 cells/cm^2^ and siRNA-mediated knockdown of FKBP10 was performed as described above. To reach 100% confluency, cells were grown for 48h followed by starvation for 24h. A scratch was executed using a 1000 μL pipette tip and TGF-β1 was added. Images were taken at time point 0h using an inverse microscope (Primovert, Carl Zeiss, Jena, Germany) and the section was marked by a black dot. After 24h, additional images were taken to compare wound closure between control and FKBP10 deficient cells. Results are given in percent of wound closure normalized to untreated control.

### Single Cell Migration Assay Using Time Lapse Microscopy

Cells were seeded at a density of 5.500 cells/cm^2^ on uncoated, collagen I-coated (~50 μg/mL, Sigma-Aldrich, St. Louis, USA), or collagen VI-coated (10 μg/mL, Abcam, Cambridge, UK) wells. Knockdown by siRNA of FKBP10 was performed as described above. After 24h, cells were serum-starved for another 24h followed by TGF-β1 treatment (2 ng/mL) for 24h. Movies were generated over a period of 12h - 24h using Axio Observer Z1 microscope equipped with an AxioCam camera (Carl Zeiss, Jena, Germany) and images were taken in 20 min intervals. Single cell movement was analyzed using the cell tracking tool of the AxioVision 4.8 software (Carl Zeiss, Jena, Germany).

## Results

### Deficiency of FKBP10 attenuates migration and adhesion of primary human lung fibroblasts

**Fig. 1:**
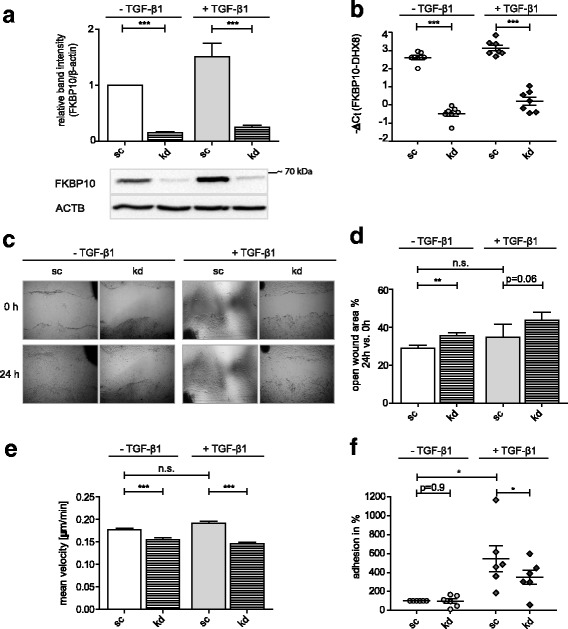
Knockdown of FKBP10 reduces migration and adhesion of phLF. **a** Western Blot analysis of phLF treated with scrambled siRNA as control (sc) or FKBP10 siRNA (kd) and 2-phosphoascorbate (0.1 mM) in absence and presence of TGF-β1 (2 ng/mL) for 48h. Densitometric analysis and representative blots show the effect of FKBP10 knockdown on the expression of FKBP10 relative to β-actin as loading control (ACTB). **b** Quantitative reverse transcriptase-polymerase chain reaction analysis of phLF treated with sc siRNA as control or FKBP10 siRNA (kd) and 2- phosphoascorbate (0.1 mM) in absence and presence of TGF-β1 (2 ng/mL) for 48h. Transcript levels are shown as -ΔCt values. DEAH (Asp-Glu-Ala-His) Box Polypeptide 8 (DHX8) was used as endogenous control. Data (**a**, **b**) is based on eight independent experiments. **c** Representative images of a scratch assay of phLF treated with sc siRNA as control or FKBP10 and 2- phosphoascorbate (0.1 mM) in absence or presence of TGF-β1 (2 ng/mL). Images were taken at 0h and after 24h. **d** Analysis of open wound areas as shown in (**c**) normalized to controls at 0h (100%), given in % of the remaining wound area. Data is based on four independent experiments. **e** SCM assay of phLF treated with sc siRNA as control or FKBP10 siRNA and 2- phosphoascorbate (0.1 mM) in absence and presence of TGF-β1 (2 ng/mL). Cells were tracked over a period of 12h - 24h. Results of five independent experiments are shown as mean velocity of around 80 tracked cells per condition. **f** Cell attachment assay of phLF treated with sc siRNA as control or FKBP10 siRNA in absence or presence of TGF-β1 (2 ng/mL) for 48h. Results originate from six independent experiments and are visualized as percentage of cell adhesion normalized to non TGF-β1-treated cells. Statistical significance between control and FKBP10 kd is indicated by horizontal brackets and asterisks

SiRNA-mediated knockdown of FKBP10 expression in phLF was highly efficient on both protein and mRNA level (Fig. 1a, b, knockdown efficiency 86% ± 5%). Wound closure in a conventional scratch assay was significantly reduced in FKBP10-deficient cells compared to control in absence of TGF-β1. A similar trend was observed in presence of TGF-β1, which just failed significance (Fig. 1c, d). These results were confirmed with an independent and more accurate method, namely a single cell migration assay using time-lapse microscopy to track individual cells. In this assay, FKBP10-deficient cells showed a highly significant reduction of mean velocity both in absence and presence of TGF-β1 (Fig. 1e).

Next, we investigated the effect of siRNA-mediated knockdown of FKBP10 on adhesion of phLF using an IF-based attachment assay. In presence of TGF-β1, FKBP10 deficiency significantly reduced the ability of phLF to adhere to the cell culture dish surface (Fig. 1f).

While TGF-β1 had no significant effect on migration, neither in the scratch nor in the single cell tracking assay, TGF-β1 significantly increased fibroblast adhesion.

### Deficiency of FKBP10 upregulates expression of key molecules of adhesion and migration in primary human lung fibroblasts, but does not alter the activation of focal adhesion kinase and downstream pathways

**Fig. 2:**
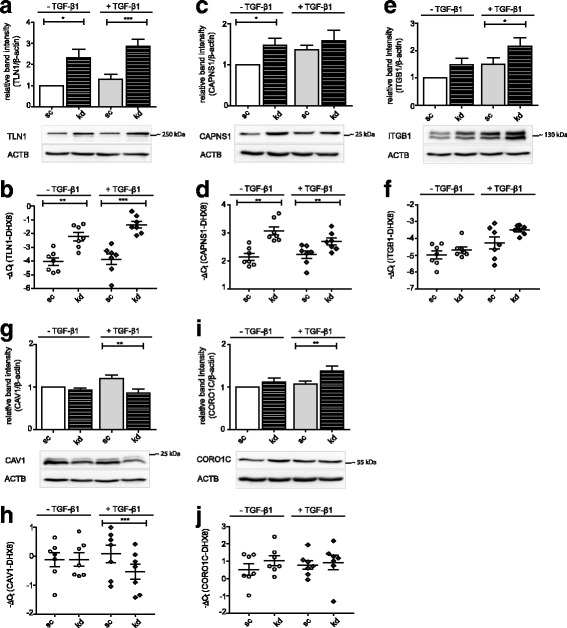
FKBP10 deficiency alters the expression of molecules implicated in adhesion and migration. **a, c, e, g, i** Western Blot analysis of phLF treated with scrambled siRNA as control (sc) or FKBP10 siRNA (kd) and 2- phosphoascorbate (0.1 mM) in absence and presence of TGF-β1 (2 ng/mL) for 48h. Densitometric analysis and representative blots show the effect of FKBP10 kd on the expression of talin-1 (TLN1) (**a**), calpain-4 (CAPNS1) (**c**), integrin β1 (ITGB1) (**e**), caveolin-1 (CAV1) (**g**) and coronin 1C (CORO1C) (**i**) relative to β-actin as loading control (ACTB). **b, d, f, h, j** Quantitative reverse transcriptase-polymerase chain reaction analysis of phLF treated with scrambled siRNA as control (sc) or FKBP10 siRNA (kd) and 2- phosphoascorbate (0.1 mM) in absence and presence of TGF-β1 (2 ng/mL) for 48h. Transcript levels are presented as -ΔCt values showing the effect of siRNA mediated kd of FKBP10 on talin-1 (TLN1) (**b**), calpain-4 (CAPNS1) (**d**), integrin β1 (ITGB1) (**f**), caveolin-1 (CAV1) (**h**) and coronin 1C (CORO1C) (**j**). DEAH (Asp-Glu-Ala-His) Box Polypeptide 8 (DHX8) was used as endogenous control. All data is based on eight (protein) or seven (mRNA) independent experiments. Statistical significance between control and FKBP10 kd is indicated by horizontal brackets and asterisks

To gain a better understanding of the effect of FKBP10 deficiency on adhesion and migration, we analyzed several selected proteins with key functions in these processes. In terms of altered gene expression of focal adhesion and ECM components, we only observed marginal differences between fibroblasts isolated from normal histology control or from IPF lung tissue and therefore decided to pool the data. Deficiency of FKBP10 in phLF led to induction of the focal adhesion component talin-1 (TLN1) on protein and mRNA level in absence and presence of TGF- β1 (Fig. 2a, b). Next, we investigated the effect of FKBP10 knockdown on calpain-4 (CAPNS1), the small regulatory subunit of calpain-1 and calpain-2, which is indispensable for formation and strengthening of adhesions and for mechanosensing during fibroblast migration [24]. Similar to TLN1, CAPNS1 expression was significantly upregulated in absence and presence of TGF-β1 on transcript level (Fig. 2c, d); this effect, however, translated to the protein level only in absence of TGF-β1. Moreover, FKBP10 knockdown also increased protein levels of the transmembrane collagen receptor integrin-β1 (ITGB1), particularly in presence of TGF-β1 (Fig. 2e), while transcript levels were not significantly changed (Fig. 2f). Finally, two modulators of cytoskeleton dynamics, caveolin-1 (CAV1) and coronin-1C (CORO1C), were regulated in opposite directions: Whereas CAV1 expression (protein and mRNA) was downregulated upon FKBP10 knockdown in presence of TGF-β1 (Fig. 2g, h), CORO1C protein levels were upregulated (Fig. 2i, j).

FAK activation by either integrins or growth factors leads to autophosphorylation of Tyr 397 in FAK, creating a motif which results in binding and conformational activation of proto-oncogene tyrosine-protein kinase Src. Active Src triggers further FAK phosphorylation on Tyr sites like Tyr 576/577, starting the ras-raf-MEK-ERK signal transduction cascade which is implicated in many processes including cell adhesion and migration. MEK1/2 catalyzes ERK1/2 phosphorylation on specific Tyr and Thr residues essential for enzyme activity [25, 26].

**Fig. 3:**
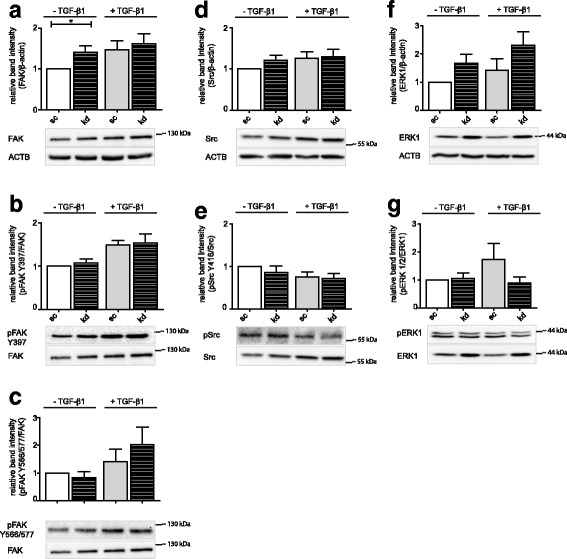
Neither FAK, Src, nor ERK1/2 activation is affected by FKBP10 kd. Western Blot analysis of phLF treated with scrambled siRNA as control (sc) or FKBP10 siRNA (kd) and 2- phosphoascorbate (0.1 mM) in absence and presence of TGF-β1 (2 ng/mL) for 48h. Densitometric analysis and representative blots show the effect of FKBP10 kd on the expression of FAK (**a**), Src (**d**) and ERK1 (**f**) relative to β-actin as loading control (ACTB). **b, c** Densitometric analysis and representative blots showing the effect of FKBP10 kd on the phosphorylation of FAK (Y397) (**b**) and FAK (Y576/577) (**c**) relative to FAK. **e, g** Densitometric analysis and representative blots showing the effect of FKBP10 kd on the phosphorylation of Src (Y416) (**e**) relative to Src and pERK1/2 (T202/Y204) (**g**) relative to ERK1. All data is based on eight independent experiments. Statistical significance between control and FKBP10 kd is indicated by horizontal brackets and asterisks

Deficiency of FKBP10 slightly upregulated total FAK levels (Fig. 3a); however, there was no significant effect on the phosphorylation on Tyr 397 or Tyr 576/577 of FAK (Fig. 3b, c). Similarly, none of the assessed downstream signaling pathways appeared affected: Neither levels of total Src and phospho-Src nor levels of total ERK1 and phosphorylated ERK1/2 changed upon FKBP10 knockdown (Fig. 3d-g).

### FKBP10 interacts with type VI collagen and fibulin-1 and regulates their expression

**Fig. 4:**
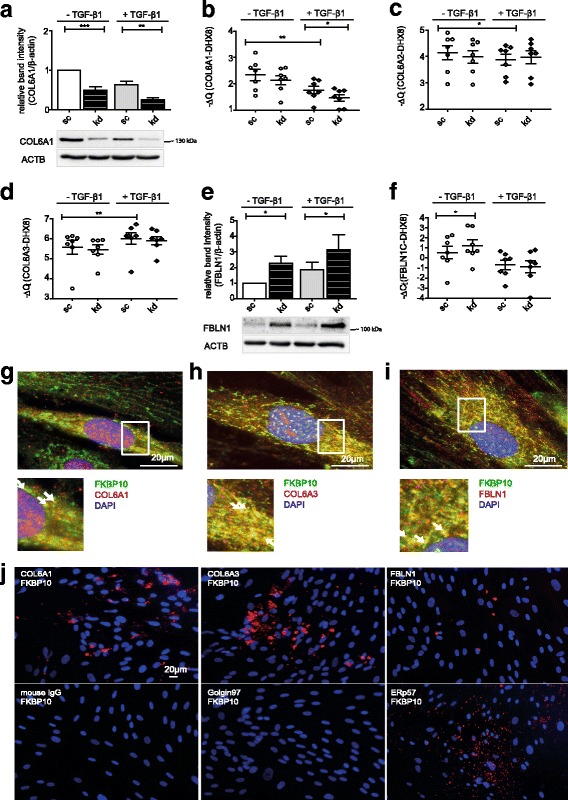
FKBP10 interacts with COL6A1 and FBLN1 and regulates their expression. **a, e** Western Blot analysis of phLF treated with scrambled siRNA as control (sc) or FKBP10 siRNA (kd) and 2- phosphoascorbate (0.1 mM) in absence and presence of TGF-β1 (2 ng/mL) for 48h. Densitometric analysis and representative blots showing the effect of FKBP10 kd on the expression of Collagen VI alpha1 (COL6A1) (**a**) and fibulin-1 (FBLN1) (**e**) relative to β-actin as loading control (ACTB). **b, c, d, f** Quantitative reverse transcriptase-polymerase chain reaction analysis of phLF treated with sc siRNA as control or FKBP10 siRNA and 2-phosphoascorbate (0.1 mM) in absence and presence of TGF-β1 (2 ng/mL) for 48h. Transcript levels are shown as -ΔCt values of showing the effect of FKBP10 kd on collagen VI alpha 1 (COL6A1) (**b**), collagen VI alpha 2 (COL6A2) (**c**), collagen VI alpha 3 (COL6A3) (**d**) and fibulin-1C (FBLN1C) (**f**). DEAH (Asp-Glu-Ala-His) Box Polypeptide 8 (DHX8) was used as endogenous control. All data is based on eight (protein) or seven (mRNA) independent experiments. Statistical significance is indicated by horizontal brackets and asterisks. **g-i** Immunofluorescence staining of FKBP10 (green) and COL6A1 (red) (**g**), COL6A3 (red) (**h**) and FBLN1 (red) (**i**). 4`, 6-diamidino-2-phenylindole (DAPI) staining is shown in blue. The region of interest is indicated by a white square and enlarged in the picture below. White arrows specify examples for co-localization of FKBP10 with COL6A1, COL6A3 or FBLN1. Stainings were taken as z-stack and the enlarged pictures show one focal plane from this z-stack. Representative images were selected from three independent experiments. **j** Representative images of *in situ* localization of the interaction of FKBP10 and COL6A1, COL6A3, FBLN1, mouse IgG1κ (negative control), Golgin97 (negative control) and ERp57 (positive control), assessed by proximity ligation assay. Representative images were selected from three independent experiments, except for IgG negative control (n=1)

As changes in expression of focal adhesion complex components and in events during FAK signaling did not explain the observed attenuated migration upon FKBP10 knockdown, we reasoned that changes in ECM composition could be the main cause of reduced migration velocity. Besides the ECM proteins collagen I and FN, which we reported to be downregulated upon FKBP10 knockdown previously [22], collagen VI and fibulin-1 also have been described to maintain important roles in the process of cell migration [18, 27]. Here, we found that collagen VI and fibulin-1 expression were regulated in different directions in response to FKBP10 knockdown. Collagen 6A1 was significantly reduced on protein level in FKBP10 deficient phLF with and without TGF-β1, whereas *COL6A1* transcript was only reduced in presence of TGF-β1 (Fig. 4a, b). Interestingly, mRNA levels of *COL6A2* and *COL6A3* chains were not influenced by FKBP10 deficiency (Fig. 4c, d). In contrast, fibulin-1 protein expression was increased in response to FKBP10 knockdown in absence and presence of TGF-β1; transcript levels of *FBLN1C* were significantly upregulated under basal conditions upon FKBP10 knockdown (Fig. 4e, f).

Co-localization of FKBP10 with both, collagen VI (COL6A1 and COL6A3) (Fig. 4g, h) and fibulin-1 (Fig. 4i) was examined by IF stainings and PLA (Fig. 4j). Negative controls (mouse IgG and Golgin97) did not show any interaction with FKBP10. Endoplasmic Reticulum Protein 57 (ERp57), previously reported by IF to co-localize with FKBP10 [22] was used as positive control and showed proximity to FKBP10. Positive signals were also observed for COL6A1, COL6A3 and FBLN1, indicating interaction with or proximity to FKBP10.

### The effects of FKBP10 deficiency on migration and adhesion depend on 2-phosphoascorbate and are abolished by coating cell culture dishes with collagen VI

**Fig. 5:**
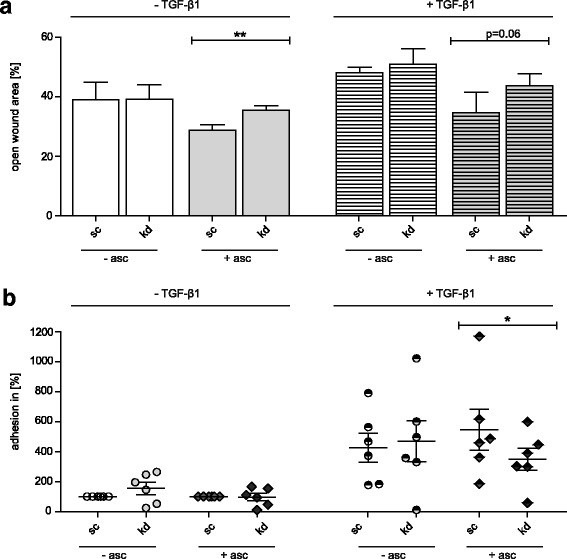
The inhibitory effect of FKBP10 deficiency on migration and adhesion is 2-phosphoascorbate dependent. **a** Analysis of open wound areas in scratch assays of phLF treated with scrambled siRNA (sc) as control or FKBP10 siRNA (kd) in absence and presence of TGF-β1 (2 ng/mL) and 0.1 mM 2-phosphoascorbate. Images were taken at 0h and after 24h. Data is normalized to controls at 0h (100%), given in % of the remaining wound area and based on four independent experiments. **b** Cell attachment assay of phLF treated with scrambled siRNA (sc) as control or FKBP10 siRNA (kd) in absence or presence of TGF-β1 (2 ng/mL) and 2-phosphoascorbate (0.1 mM) for 48h. Results originate from six independent experiments and are visualized as percentage of cell adhesion normalized to non TGF-β1 treated. Experiments without and with 2- phosphoascorbate were performed in parallel

Post-translational modifications (PTM) like proline and lysine hydroxylation of collagens (including collagen VI) are crucial steps in collagen biosynthesis and catalyzed by ascorbate-dependent prolyl and lysyl hydroxylases [28–30]. In contrast, FBLN1 biosynthesis is not dependent on ascorbate [31]. Therefore, we reasoned that exclusion of ascorbate from the culture medium would give us a first indication whether decreased biosynthesis of collagen or increased biosynthesis of fibulin may underlie the observed effects of FKBP10 deficiency on migration and adhesion. Notably, in absence of 2-phosphoascorbate the effect of FKBP10 deficiency in phLF on adhesion and migration was lost, in comparison to the results in presence of 2-phosphoascorbate (Fig. 5), pointing towards a collagen-dependent mechanism.

**Fig. 6:**
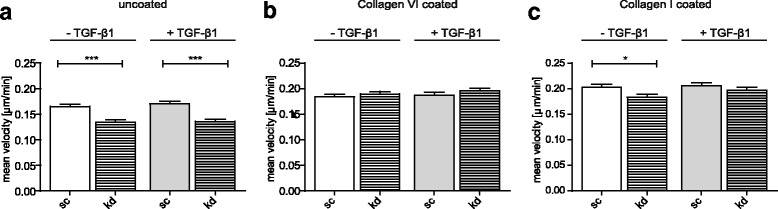
Coating of dishes with collagen VI abolishes the inhibitory effect of FKBP10 knockdown on migration. SCM assay of phLF treated with scrambled siRNA as control (sc) or FKBP10 siRNA (kd) and 2- phosphoascorbate (0.1 mM) in absence and presence of TGF-β1 (2 ng/mL). Cells were tracked over a period of 12h -24h. Results of three independent experiments are shown as mean velocity of around 80 tracked cells per condition. Effect of collagen VI coated dishes on mean velocity of phLF is shown in (**b**) and of collagen I coated dishes in (**c**) compared to uncoated dishes for control (**a**). Statistical significance between control and FKBP10 kd is indicated by horizontal brackets and asterisks

As both collagen I and collagen VI are downregulated upon FKBP10 deficiency [22], we coated dishes with either collagen I or collagen VI and analyzed migration in a single-cell migration approach. Notably, the effect of FKBP10 deficiency in phLFs on migration was completely lost when culture dishes were coated with collagen VI (Fig. 6b) when compared to uncoated dishes (Fig. 6a). Some compensation was also visible in collagen I-coated dishes, albeit not that pronounced (Fig. 6c).

## Discussion

In this study we demonstrated that FKBP10 deficiency inhibited phLF adhesion and migration. This effect could neither be explained by changes in expression or activation of components of the FA complex, nor by changes in FAK downstream signaling events, nor by changes in regulators of actin dynamics. Instead, we found that the effect of FKBP10 deficiency on migration and adhesion depended on 2-phosphoascorbate, pointing towards a central role of collagen biosynthesis in this context. Loss of FKBP10 downregulated the expression of collagen VI, a collagen type increasingly recognized as a central player in migration, and coating culture dishes with collagen VI completely abolished the effect of FKBP10 deficiency on phLF migration.

Next to excessive ECM deposition by interstitial fibroblasts, aberrant fibroblast adhesion and increased migration are also important features of IPF [6, 9–11]. Here we show that loss of the collagen chaperone FKBP10, which we previously identified as potential IPF drug target due to its role in ECM synthesis and secretion [22], inhibited wound closure and reduced the mean velocity and adhesion capacity of phLF (Fig. 1). This observation is in line with the very recent report by Liang *et al.*, showing that siRNA-mediated knockdown of FKBP10 led to reduced migration in human hypertrophic scar fibroblasts [32]. Collectively, these studies are confirmative of our concept of FKBP10 as potential drug target in fibrotic disease.

We assessed fibroblast migration both in a conventional scratch assay as well as by tracking individual cells with videomicroscopy in time-lapse experiments, a more accurate approach. Overall, both assays consistently showed reduced migration under conditions of FKBP10 deficiency, even if the scratch assay results in presence of TGF-β1 failed significance (p=0.06). We believe that this minor discrepancy reflects the well-known disadvantages of the scratch assay, most importantly variations in gap width due to the manually applied and therefore often uneven scratch, the fact that ECM substrate is equally scraped off together with the cells, and also the mechanical cell damage which may introduce artifacts. These technical drawbacks may explain the greater variations observed in this assay, leading to results that just fail significance.

Our finding that TGF-β1 did not affect fibroblast migration is in agreement with previous studies by others [33–35]. Increased fibroblast adhesion in presence of TGF-β1 may in part reflect increased expression of β1-integrin (this work, cf. Fig. 2e, f, scr ctrl *vs*. scr TGF-β1, p=0.0642) and/or β3-integrins [36].

Initially, we sought to assess the effect of FKBP10 deficiency on several regulatory levels of FA turnover to elucidate the mechanism underlying attenuated migration. Cell migration is a complex cyclic process starting with the extension of membrane protusions (*lamellipodia*) at the leading edge followed by their adhesion to the ECM and retraction of the cell tail [37]. During cell migration assembly and disassembly of FA is dynamically regulated. The process of cell attachment to the ECM is initiated by clustering of integrins on the cell surface, heterodimeric transmembrane receptors consisting of α and β subunits. The intracellular domains of the clustered integrins serve as a platform for FA protein recruitment and ultimately link the ECM via FA to actin stress fibers [38]. In this context, TLN1 mediates the initiation of FA assembly by interacting with the cytoplasmic domain of the integrin β subunit on the one hand and with actin and actin-binding proteins on the other [15]. Another initial event upon integrin clustering is activation of FAK including autophosphorylation of Tyr 397 and Src-mediated phosphorylation of additional tyrosine residues within FAK (*e.g.* Y 566 and 577), which are essential for full FAK activity. Active FAK interacts with multiple signalling molecules including Grb7, PI3K, paxillin, MLCK and ERK, activating signalling pathways which result in protrusion extension, increased FA turnover, and therefore, ultimately, in increased cell motility [15, 39–42]. The protease calpain 2, a heterodimer consisting of a catalytic subunit and a regulatory subunit (calpain-4, CAPNS1) plays a central role in this context as it, when recruited by FAK and activated by ERK/MAP kinase, mediates amongst others proteolysis of TLN1 and FAK, considered the rate-limiting step in FA turnover [15, 38, 39, 43, 44]. Finally, directional cell migration is strongly dependent on polarized actin dynamics. Rho-like GTPases like RhoA and Rac1 control cytoskeleton contractility, polymerization, and protrusion. The integral membrane protein caveolin-1 (Cav-1), activated by small kinases like Src, is a central regulator of Rho-like GTPase signaling in this context [45, 46]. Another regulator of actin filament turnover in the *lamellipodia* during cell migration is the actin binding protein coronin 1C (CORO1C) [47].

FKBP10 deficiency led to several significant changes in expression of FA components on all levels assessed, *i.e.* upregulation of ITGB1, TLN1, CAPNS1, FAK, CORO1C and downregulation of CAV1 (Fig. 2). Upregulation of ITGB1 may associate with reduced FA turnover and slow migration [48–50] but impaired migration and adhesion has also been reported as a result of ITGB1 deficiency [51]. Collectively, these findings suggest that manipulation of ITGB1 protein levels in general is unfavorable to cell motility, regardless of direction of regulation. As ITGB1 functions as an adapter protein between the ECM and intracellular FA complexes, it is conceivable that its expression levels must be tightly regulated to allow for functional intermolecular interactions between different interacting partners.

The same may apply to TLN1, an adapter protein linking the cytoskeleton to ITGB1, where reports in the literature are also seemingly controversial: Upregulation of TLN1, but also suppression of TLN1 has been reported to reduce migration and adhesion [52–54]. Interestingly, downregulation of TLN1 has also been associated with increased migration [52, 55], which is in support of its function as a regulator of FA turnover together with its protease calpain-2 [15, 38, 39, 43, 44]. In our system, however, the simultaneous increase of the regulatory calpain 2 subunit CAPNS1, argues for overall little change in FA turnover, at least in absence of TGF-β1 (Fig. 2c, d). This is consistent with our observation that phosphorylation levels of FAK, Src, and ERK (Fig. 3), central signaling events in the process of FA turnover [39, 56–58] remained unchanged.

At first, observing inhibition of adhesion and migration in the absence of changes in activation of FAK and related signaling pathways seemed contradictory to us. However, similarly, Asano and colleagues have reported that siRNA-mediated knockdown of α-smooth muscle actin (α-SMA) in phLF led to inhibition of migration without affecting the FAK signaling pathway [59]. This observation suggested that changes in actin dynamics may underlie the observed inhibition of migration and, indeed, from our previous studies, we know that FKBP10 deficiency reduces α-SMA expression in phLF [22]. Also, deficiency of the actin binding protein CORO1C typically results in inhibition of migration; however, here, we observed a moderate increase of CORO1C protein rather than downregulation (Fig. 2i) [60, 61]. Expression of CAV1, deficiency of which typically results in decreased migratory speed in variable cell types [62–64], was only moderately downregulated in presence of TGF-β1 (Fig. 2g, h). Collectively, these observations do not argue for altered actin dynamics as a major mechanism underlying inhibition of migration in response to FKBP10 deficiency.

Importantly, cell migration is influenced by properties of the ECM, like density of adhesion ligands (collagen, FN), ECM composition, and stiffness [7, 65]. Previously, we have observed downregulation of expression and secretion of type I collagen and FN, both major components of the fibroblast ECM, in response to FKBP10 knockdown [22]. Here we extended this analysis and assessed additional ECM components with important roles in cell migration, namely type VI collagen and FBLN1. Both proteins colocalized with FKBP10 in phLF, as assessed by both immunofluorescence colocalization analysis and proximity ligation assay (Fig. 4g-j), indicating direct interaction with FKBP10 in the ER. Interestingly, we found that loss of FKBP10 significantly increased FBLN1 expression (Fig. 4e, f), but decreased protein levels of COL6A1 (Fig. 4a, b). Notably, COL6A1 deficiency is sufficient to inhibit collagen VI deposition in the ECM, as no triple helical molecules can be formed without the α1(VI)-chain [66]. These results suggest opposing functions of FKBP10 in FBLN1 and collagen VI biosynthesis in phLF. It is tempting to speculate, for instance, that FKBP10 acts as a FBLN1 chaperone, sequestering FBLN1 in the ER, prohibiting packing in vesicles for secretion or targeting FBLN1 for ER-associated protein degradation, while at the same time FKBP10 is likely required for efficient collagen VI triple helix formation, similar to collagen I and III [21, 67, 68]. These aspects will be interesting to explore in future studies.

As to function of these proteins in migration, reduced attachment and decreased migratory speed has been reported for a human breast cancer cell line (MDA MB231) in response to FBLN1 overexpression [27] and siRNA mediated knockdown of FBLN1 in corneal fibroblasts upregulated cell migration [69]. Therefore, taken together, it was plausible that increased FBLN1 levels may underlie the observed inhibition of migration under conditions of FKBP10 deficiency.

While collagen VI begins to emerge as an important regulator of cell migration, reports on its direction of effect, inhibiting or promoting migration, are controversial [18–20]. For instance, collagen VI-deficient tendon fibroblasts show delayed wound closure, *i.e.* lower migration speed, in a scratch assay [18], while human dermal fibroblasts displayed higher migration speed on matrices derived from collagen VI-deficient cells [20]. These discrepancies may be a result of the divergent approaches in the mentioned studies (assessment of newly formed ECM versus assessment of ECM deposited within 10 days, respectively), different collagen VI chains assessed (COL6A1 versus COL6A2), but also of the different cell origins, thus possibly reflecting time-, chain-, and cell-specific effects of collagen VI.

Ascorbic acid is a cofactor necessary for proline and lysine hydroxylation during collagen synthesis [68] including collagen VI [30, 70], but not required for FBLN1 synthesis. Therefore, to differentiate between increased FBLN1 or decreased type VI collagen as the underlying mechanism of decreased adhesion and migration, we compared effects of FKBP10 knockdown on adhesion and migration in absence and presence of 2-phosphoascorbate, a stable analogue of ascorbic acid. Notably, neither migration nor adhesion were changed upon loss of FKBP10 when the cell culture medium was ascorbate-deficient (Fig. 5a, b). These results strongly indicated that the effect of FKBP10 deficiency on adhesion and migration was collagen-dependent. While coating with collagen VI abolished the effect of FKBP10 knockdown on migration completely, coating with collagen I only did so marginally (Fig. 6). Overall, this indicated that FKBP10 knockdown inhibits lung fibroblast migration by reduced collagen VI biosynthesis rather than reduced collagen I biosynthesis, an effect of FKBP10 deficiency which we have reported previously [22].

## Conclusion

Collagen VI is an important regulator of the ECM organizing the three-dimensional meshwork of collagen and FN fibers [71, 72] and the topography of the fibrillar ECM network guides directional cell migration [73]. Therefore, our observations suggest that loss of FKBP10 results in reduced biosynthesis of collagen VI and, possibly in combination with decreased extracellular collagen I levels, leads to a disorganized ECM with changed adhesion ligand density, stiffness and composition, which may not provide sites for integrin clustering and does not favor directional cell migration. Upregulation of ITGB1, TLN1, CAPNS1, total FAK, and CORO1C may ultimately reflect an attempt of the cells to overcome decreased migration by overcompensation, increasing expression of ECM receptors or components of the FA turnover machinery.

Interestingly, FKBP10 mediates dimerization with collagen lysyl hydroxylase 2 and thus contributes to the generation of collagen hydroxylysines, which act as substrates for extracellular lysyl-oxidase-mediated collagen crosslinking [74–76]. Extracellular collagen lacking proper crosslinks is prone to proteolytic degradation and may not be able to contribute to the higher ordered organization of the ECM anymore [77–80]. Therefore, downregulation of FKBP10 may also contribute to disorganization of the ECM by providing less crosslinking sites in type I and type VI collagen.

In summary, we found that loss of FKBP10 in phLF results in decreased adhesion and migration. We found that the main underlying mechanism was reduced collagen VI biosynthesis, as both ascorbate deficiency and coating of cell culture dishes with collagen VI abolished the effect of FKBP10 knockdown on migration. As increased fibroblast migration is a characteristic of IPF, the results are in support of our concept of FKBP10 as a potential drug target for IPF.

## Abbreviations

FKBP10: FK506-binding Protein 10
IPF: Idiopathic Pulmonary Fibrosis
ECM: Extracellular matrix
phLF: Primary Human Lung Fibroblast
TGF-β1: Transforming Growth Factor-β1
IF: Immunofluorescence
FAK: Focal Adhesion Kinase
FA: Focal Adhesions
Src: SRC Proto-Oncogene, Non-Receptor Tyrosine Kinase
ERK: Extracellular Signaling Related Kinase
MAP: Mitogen-activated Protein
FN: Fibronectin
ER: Endoplasmic Reticulum
α-SMA: α-smooth muscle actin
ACTB: β-actin
CAPNS1: Calpain-4
CAV1: Caveolin-1
COL6A1: Collagen VI α1
COL6A3: Collagen VI α3
CORO1C: Coronin 1C
ERp57: ER protein 57 (Protein disulfide-isomerase A3)
FBLN1: Fibulin-1
ITGB1: Integrin-β1
TLN1: Talin 1
COL6A1: Collagen VI alpha 1
COL6A2: Collagen VI alpha 2
COL6A3: Collagen VI alpha 3
PTM: Post-translational Modification
sc: scrambled
kd: knockdown
